# Using CT-guided stereotactic prostate radiation therapy (CT-SPRT) to assess sustained murine prostate ablation

**DOI:** 10.1038/s41598-021-86067-8

**Published:** 2021-03-22

**Authors:** Ali H. Zahalka, N. Patrik Brodin, Maria Maryanovich, Xizhe Wang, Kara L. Watts, Sandra Pinho, Chandan Guha, Paul S. Frenette

**Affiliations:** 1 Department of Urology, Albert Einstein College of Medicine/Montefiore Medical Center, Bronx, NY USA; 2 Department of Cell Biology, Albert Einstein College of Medicine, 1301 Morris Park Ave, Bronx, NY 10461 USA; 3 Department of Radiation Oncology, Albert Einstein College of Medicine/Montefiore Medical Center, Bronx, NY USA; 4 Department of Genetics, Albert Einstein College of Medicine, Bronx, NY USA; 5 Ruth L. Gottesman Institute for Stem Cell and Regenerative Medicine Research, Albert Einstein College of Medicine, Bronx, NY USA; 6 Department of Pharmacology and Regenerative Medicine, University of Illinois at Chicago, Chicago, IL USA; 7 Department of Medicine, Albert Einstein College of Medicine, Bronx, NY USA; 8 Present Address: Department of Urology, Icahn School of Medicine at Mount Sinai, One Gustave L. Levy Place, Box 1272, New York, NY 10029 USA

**Keywords:** Urogenital models, Prostate, Urogenital diseases, Experimental models of disease, Preclinical research

## Abstract

The prostate is a hormone-responsive organ where testicular androgens drive the proliferation and survival of prostatic cells, ensuring the development and functioning of this gland throughout life. Androgen deprivation therapy leads to apoptosis of prostatic cells and organ regression, and is a cornerstone of prostate cancer and benign prostatic hypertrophy treatment. For several decades, androgen deprivation has been used as an adjuvant to external beam radiotherapy, however, emerging data suggests that the low rates of epithelial proliferation in the castrated prostate imparts radio-resistance. As proliferating cells exhibit increased sensitivity to radiation, we hypothesized that short bursts of synchronized epithelial proliferation, which can be achieved by exogeneous testosterone supplementation prior to targeted high-dose radiation, would maximize sustained prostate ablation, while minimizing damage to surrounding tissues. To test this hypothesis, we designed a novel computed-tomography (CT)-guided stereotactic prostate radiation therapy (CT-SPRT) technique to deliver a single high-dose 25 Gy fraction of X-ray radiation. Sustained prostatic cell ablation was assessed post CT-SPRT by measuring prostate weight, epithelial cell number, and relative contributions of luminal and basal epithelial populations in control and testosterone-pretreated glands. CT-SPRT was safely delivered with no observed damage to surrounding rectal and bladder tissues. Importantly, castrated mice that received a pulse of testosterone to induce synchronous cell proliferation prior to CT-SPRT exhibited significant sustained gland ablation compared to control mice. These results provide new insights in stereotactic radiotherapy sensitivity to maximize prostatic cell ablation and improve our understanding of prostate gland regeneration that can potentially lead to improved non-invasive therapies for benign prostatic hypertrophy and prostate cancer.

## Introduction

External beam radiation therapy is one of the main modalities for definitive prostate cancer treatment. This modality is finding increasing therapeutic and survival benefits in the treatment of prostate cancer including localized disease, localized high-risk disease, and as salvage therapy in both localized-recurrent as well as metastatic disease^1–4^. Radiation causes cell death by inducing unrepairable double-strand DNA breaks, preferentially affecting actively proliferating cells^5^. However, radiation dose and the number of doses, or fractions, delivered to the tissue is in part dependent on the differential radiation sensitivity (related to cellular proliferative index) of the prostate tumor and the surrounding non-malignant tissues^6^.

A growing body of evidence suggests that the radiosensitivity of the prostate and the surrounding tissues are similar^7^. Thus, to minimize off-target effects and damage to surrounding healthy tissues, precision techniques utilizing image-guided radiation, known as stereotactic body radiation therapy (SBRT), are clinically employed to deliver very high doses of radiation in as few fractions (hypofractionation) as possible^8^.

As men age, they are at increased risk for prostate cancer as well as urinary obstruction from benign prostatic hypertrophy (BPH)^9^. This benign, but highly bothersome condition is histologically characterized by benign prostatic epithelial cell growth. BPH and prostate cancer are often found together in the prostates of elderly men^9^. While radiation therapy has been shown to have equivalent oncologic outcomes as surgical therapy^10^, it has limited gland ablative effects, requiring further intervention for comorbid BPH including surgical resection of the BPH tissue to relieve urinary obstruction^11^.

While the prostate epithelium has a low baseline rate of proliferation, and thus similar radiosensitivity as the surrounding tissues, the presence of testosterone (the main androgen in the prostate) can selectively increase prostate epithelial cell proliferation 200-fold^12^. Similarly, prostate epithelial cells are also extremely sensitive to the withdrawal of androgens, with greater than 90% of luminal epithelial cells undergoing apoptosis following castration-induced androgen deprivation^13^. However, after exogenous testosterone supplementation, the castrated prostate regenerates back to its initial size within 2 weeks^14^. This rapid regeneration is due to a proliferative burst of androgen-deprived prostate epithelium in castrated mice (henceforth referred to as castrate epithelium) in response to testosterone^13^. As proliferating cells exhibit increased sensitivity to radiation, we hypothesized that a short course of testosterone supplementation prior to irradiation would increase the gland-ablative effects of SBRT.

Due to its small size and lack of enhancement on computed-tomography (CT), the murine prostate is difficult to distinguish from surrounding structures, and thus cannot be stereotactically targeted for high-dose X-ray irradiation by conventional imaging techniques. We therefore developed a novel technique that employs contrast enhanced CT-guided imaging and targeted X-ray irradiation of the mouse prostate that we termed stereotactic prostate radiation therapy (CT-SPRT). We then employed this novel technique to study the effect of radiation on the regenerating prostate. In this study, we detail the novel CT-SPRT technique and investigate the effects of a single high-dose 25 Gy fraction of X-ray radiation on sustained prostate ablation.

## Results

### Intra-prostatic contrast injection enables CT-SPRT

**Figure 1:**
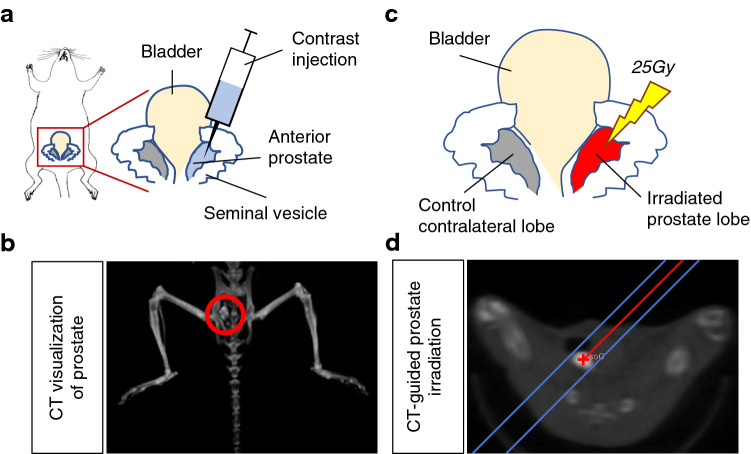
CT-guided stereotactic prostate radiation therapy (CT-SPRT) technique. (**a**) Diagram depicting intra-prostatic contrast injection to enable CT-visualization of the prostate (**b**; Left panel) and CT-guided targeting for X-ray irradiation of the prostate (**d**; Right panel); (**c**) Diagram depicting administration of a single fraction 25 Gy CT-targeted unilateral anterior prostate lobe X-ray radiation (CT-SPRT). Contralateral non-irradiated lobe acting as an internal control.

To visualize the murine prostate on CT imaging, we exposed the prostate through a small midline incision and orthotopically injected an iodinated contrast agent (Iodixanol, which is commonly used in humans) into the anterior lobe of the prostate, the largest and most easily accessible of the lobes (Fig. 1a). After contrast injection, the murine prostate appeared on CT imaging as a bright high-contrast region (Fig. 1b). Using the small animal radiation research platform (SARRT, a CT-guided targeted irradiation system), we were able to stereotactically target the contrast-labeled prostate lobe and robotically angle the X-ray radiation beam to avoid vital structures using CT image guidance (Fig. 1c). This technique enabled us to administer CT-SPRT in a single 25 Gy fraction (Fig. 1d), similar to the single fraction ultra-high dose SBRT used in human trials (ClinicalTrials.gov number, NCT02570919) with no post-radiation rectal or bladder toxicity observed in the treated animals (data not shown), thus validating our proof-of-concept technique.

### Testosterone-induced cell proliferation peaks after 48 h

**Figure 2:**
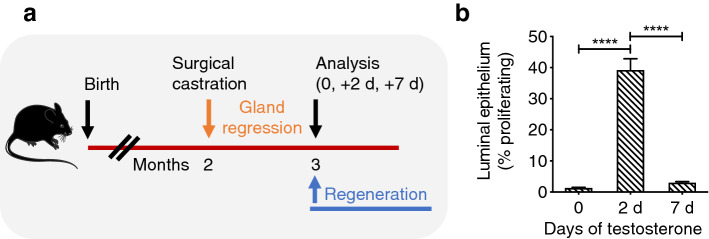
Assessment of epithelial cell cycling in the regenerating prostate. (**a**) Experimental design assessing time course of prostate epithelial cell proliferation after testosterone administration (via subcutaneous Alzet pumps). Blue lettering = periods when testosterone is present. Orange lettering = periods when testosterone is absent; (**b**) Quantification of testosterone-responsive luminal epithelial cell proliferation (by Ki67 positivity in CD24 + EPCAM + cells, quantified by FACS) post testosterone administration. n = 4 mice per condition. ****P < 0.0001. Error bars indicate SEM.

As proliferating cells exhibit increased sensitivity to radiation^5^, we next tested out the hypothesis that short bursts of synchronized epithelial proliferation prior to targeted high-dose radiation, would maximize the efficiency of CT-SPRT in sustained prostate ablation. To achieve synchronized epithelial proliferation, we adopted a model of prostate regeneration^15^ where castrated prostate epithelial cells exhibit a synchronous proliferative burst in response to testosterone supplementation^13^. First, we performed a time course (0, 2 and 7 days) to determine prostate epithelial cell proliferation kinetics in our model (Fig. 2). To accomplish this, we surgically castrated adult male mice, thus depriving the prostate epithelium of androgens, and waited 1 month for the prostate gland to regress (Fig. 2a). Prostate regeneration was stimulated by administering continuous testosterone via subcutaneous pump (to restore physiologic serum testosterone levels) in castrated mice. Luminal epithelial cells, phenotypically defined as CD45^−^, Ter119^−^, EPCAM^+^, CD24^+^, CD49f^−^^16^, are the main component of the prostate as they express high levels of the testosterone receptor^14,15,17^. We found that testosterone administration in castrated mice induced maximal luminal epithelial cell proliferation (measured by Ki67 positivity and quantified by flow cytometry) 2 days post-testosterone administration (Fig. 2b). This observed time course of maximal epithelial cell proliferation after androgen administration in castrated mice was similar to previously reported studies^12,13^. At day 2 post-testosterone administration, the percentage of Ki67^+^ proliferating epithelial cells increased from < 2% pre-androgen (castrate levels), to > 40% (testosterone pulse levels), and then back to basal (pre-castrate) levels (~ 3%) within one week with continuous testosterone administration (Fig. 2B). Thus, these results indicate that the prostate may be maximally sensitive to radiation 2-days post-testosterone pulse due to the peak in synchronous epithelial cell proliferation.

### Short pulse of testosterone prior to CT-SPRT maximizes prostate ablation

**Figure 3:**
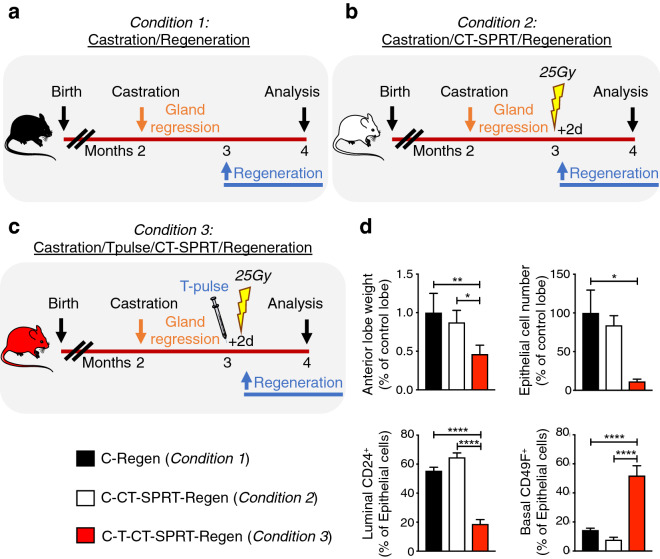
Effect of various CT-SPRT regimen on sustained prostate ablation. Diagrams depicting experimental design to assess the effect of: (**a**) Condition 1, post-castration testosterone-induced prostate regeneration; (**b**) Condition 2, effect of CT-SPRT on the castrate prostate prior to exogenous testosterone-induced regeneration; and (**c**) Condition 3, the effect of short pulse (2 days) of exogenous testosterone prior to CT-SPRT on the castrate prostate, then testosterone-induced regeneration. (**d**) Quantification of post intervention prostate weight (Top Left), epithelial cell number (Top Right), relative luminal epithelial cell (CD24^+^) contribution (Bottom Left), and relative basal epithelial cell (CD49f^+^) contribution (Bottom Right). n = 4 to 5 mice per condition. Castration-Regeneration = C-Regen (Condition 1); Castration-CT-SPRT-Regeneration = C-CT-SPRT-Regen (Condition 2); Castration-TestosteronePulse-CT-SPRT-Regeneration = C-T-CT-SPRT-Regen (Condition 3). *P < 0.05; **P < 0.01; ****P < 0.0001. Error bars indicate SEM.

The murine prostate has extensive regenerative capacity, with the ability to repeatedly regrow after more than 30 cycles of serial androgen-deprivation and replacement^18^. As hypofractionated stereotactic radiation therapy aims to maximize gland ablation, we next tested whether inducing prostate epithelial cell proliferation with a short pulse of testosterone prior to CT-SPRT can maximize sustained prostate gland ablation. As the anterior lobes of the prostate gland are paired bilateral structures (Fig. 1c), we took advantage of this anatomic symmetry to selectively irradiate one anterior lobe, while using the contralateral anterior lobe as an internal non-irradiated control (Fig. 1c). We then compared sustained prostate gland ablation (defined as reduction in gland weight and cell epithelial number after 30 days of testosterone-mediated regeneration) in three experimental conditions. Condition 1: prostate regression (androgen-deprivation by surgical castration), followed by one month of testosterone-induced gland regeneration (hereto referred to as Castration/Regeneration; see scheme in Fig. 3a). Condition 2: prostate regression, followed by unilateral irradiation of one anterior lobe of the castrated prostate with CT-SPRT of 25 Gy, and then followed by one month of testosterone-induced gland regeneration (hereto referred to as Castration/CT-SPRT/Regeneration; see scheme in Fig. 3b). Condition 3: prostate regression, followed by a 2-day pre-radiation testosterone pulse to induce prostate epithelial cell proliferation, then unilateral irradiation of one anterior lobe of the castrated prostate with CT-SPRT of 25 Gy, and then followed by one month of testosterone-induced regeneration (Castration/TestosteronePulse/CT-SPRT/Regeneration; see scheme in Fig. 3c). All experiments were proceeded by a month of testosterone induced regeneration as the prostate would remain atrophic (due to lack of testosterone-responsive luminal epithelial cells) without the regeneration period.

We found that X-ray irradiation of the castrated prostate with CT-SPRT of 25 Gy (Condition 2) did not affect subsequent gland regeneration relative to control non-irradiated gland (Condition 1), as seen by the absence of differences in lobe weight or epithelial cell numbers (Fig. 3d). However, in agreement with our initial hypothesis, inducing rapid burst of prostate epithelial cell proliferation in castrated prostates with a 2 days pulse of testosterone prior to high dose X-ray irradiation (Condition 3), had a significant gland-ablating effect, reducing subsequent gland regeneration by more than 50% compared to controls (Condition 1 and Condition 2; Fig. 3d and Supplementary Fig. 1). Furthermore, in the C-T-CT-SPRT-Regen group (Condition 3) there was a selective reduction in testosterone responsive CD24^+^ luminal epithelial cell population as compared CD49f^+^ basal cells (the non-testosterone-responsive epithelial cell population; Fig. 3d). This indicates that a short testosterone pulse prior to CT-SPRT maximizes gland ablation by selectively targeting the testosterone-responsive luminal epithelial cell population.

## Discussion

In this study, we describe a novel pre-clinical technique to visualize the mouse prostate using CT imaging and to stereotactically target it with a single high-dose 25 Gy fraction of X-ray radiation (CT-SPRT). Furthermore, this new technique enabled us to study the effects of high-dose radiation on the regenerating castration-resistant prostate. We found that a short pulse of testosterone to induce rapid prostate epithelial cell proliferation prior to CT-SPRT significantly increased sustained gland ablation and inhibited prostate regeneration, maximizing the efficacy of CT-SPRT.

As radiation therapy—especially high-dose hypofractionated stereotactic radiotherapy—is finding increasing therapeutic utility in all stages of prostate cancer treatment^1–4,19^, pre-clinical models to optimize this technique and minimize side effects are needed. Current pre-clinical techniques rely on irradiation of subcutaneously injected tumors, which do not recapitulate the native anatomical context of the prostate nor contributions of the native tumor microenvironment; or they have off-target toxicity by irradiating the entire pelvis^20^, which limits maximum radiation dose due to nearby organs at risk situated in the pelvic region. Thus, our CT-SPRT technique is the first to overcome these limitations in a pre-clinical model by specifically and reproducibly stereotactically targeting the native prostate by CT image-guided irradiation.

Increasing interest in employing high doses of radiation in few fractions stems not only from convenience for the patient (enabling fewer radiation therapy sessions in a shorter time period), but also from the radiobiology of prostate cancer that suggests high-dose hypofractionated therapy may be a more effective treatment than the current low-dose hyperfractionated standard of care^6,7^. Unlike most tumors that have a high proliferative index compared to surrounding tissues, prostatic tumors are characterized by very low numbers of proliferating cells, similar to- or lower than- surrounding tissue^21^. Therefore, the rationale behind conventional therapy where many small hyperfractionated doses of radiation are given to cause eventual cell death by inducing cumulative genetic damage over longer periods of time (late effects), may provide suboptimal prostate cancer control as well as increased toxicity to nearby healthy tissues as compared to hypofractionated high-dose strategy that induces immediate cell death by apoptosis (early effects). As a model system of the single dose hypofractionation regimen currently employed in clinical trials, we studied the effect of CT-SPRT of a single 25 Gy fraction on the regenerating castrate prostate to study contributions of early and late effects of radiation on sustained gland ablation. In the androgen-deprived castrate prostrate (exhibiting low levels of epithelial cell proliferation), we found that CT-SPRT did not have an effect on subsequent gland regeneration, as the post-irradiation regenerated gland was similar in size and cell number as non-irradiated controls. However, CT-SPRT after a short pulse of testosterone to induce maximal castrate epithelial cell proliferation, exhibited a drastic early radiation effect with reduction in both regenerated prostate gland size and epithelial cell number due to a decrease in testosterone-responsive luminal cells compared to controls. Castration-resistant androgen-responsive prostate stem cells with a luminal signature have been shown to be responsible for gland regeneration in the presence of exogenous testosterone, and are thought to be the origin of prostate cancer^22^. However, in the absence of testosterone, these stem cells are quiescent in the castrated prostate, a characteristic that imparts radioresistance^23^. As we did not see a change in immune cell populations between treatment groups, the innate immune system does not likely play a significant role in the proposed mechanism. Our findings thus suggest a potential novel radiation treatment paradigm, where a short course of testosterone is administered prior to hypofractionated irradiation (CT-SPRT) to maximize sustained gland ablation.

The gland-ablative effects of our treatment paradigm also has implications for the treatment of BPH. As the prevalence of BPH, a benign outgrowth of prostatic epithelial tissue causing urinary obstruction, and prostate cancer increase with age, these two conditions are often found together^9^. As conventional radiation therapy induces cell death in malignant prostate tissue but minimally reduces prostate size (on average 10 percent)^24^, patients with comorbid BPH often require additional intervention including surgical resection of prostate tissue to relieve urinary obstruction, which can lead to incontinence^11^. Thus, our technique may provide a novel treatment paradigm that addresses both prostate issues.

While 25 Gy is an ultra-high dose of radiation to deliver in a single fraction, it has been shown to be a safe and effective dose in treating spinal metastases^25^, and is under investigation for the treatment of localized prostate cancer (ClinicalTrials.gov number, NCT02570919). Thus, the technique and dosage that we explored has direct clinical relevance to humans. To complement our studies in the benign setting of regeneration, further studies should evaluate the impact of localized irradiation in the context of autochthonous mouse models of recurrent castration-resistant prostate cancer. Specifically, the effects of short testosterone pulse prior to CT-SPRT on rates of cancer ablation and remission in localized recurrent castration-resistant disease should be studied, as clinical studies have not shown a survival benefit using current irradiation paradigms in this most aggressive of disease stages^26^. Furthermore, the gland ablative effects of our hormone plus irradiation paradigm warrants further study as a non-invasive treatment for BPH, especially in those who are not surgical candidates.

CT-SPRT is a novel pre-clinical technique to safely and reproducibly administer ultra-high dosage of radiation to the prostate. Furthermore, short-course androgen administration (testosterone pulse) prior to CT-SPRT maximizes castrate prostate ablation and inhibits testosterone-induced regeneration in mice.

## Methods

### CT-guided stereotactic prostate radiation therapy (CT-SPRT)

This study complied with all ethical guidelines and regulations involving experiments with mice, and all experimental procedures performed on mice were approved by the Animal Care and Use Committees of the Albert Einstein College of Medicine, as well as followed the ARRIVE Guidelines 2.0. Male wild-type C57BL/6 mice undergoing CT-guided imaging and stereotactic irradiation were anesthetized with 1.5% isoflurane in pure oxygen, the abdomen depilated and sterilized, and a 1 cm midline incision was made to visualize the bladder and bilateral anterior prostate lobes. 4μL of Iodixanol contrast agent (320 mgI/mL; GE Healthcare, USA) was injected unilaterally into one of the anterior prostate lobes (Fig. 1a) using a 31gauge needle attached to a Hamilton syringe (Hamilton Corporation, USA), vital organs moved out of X-ray path, then the abdomen and skin sutured closed.

The unilateral prostate lobe to be treated was visualized as a single bright high-contrast region on a cone-beam computed tomography scan (small animal radiation research platform, SAARP, Xstrahl, Surrey, UK) with a reconstructed image resolution of 0.275 × 0.275 × 0.275 mm^3^ This high contrast region was targeted for irradiation using a 3 mm × 3 mm collimator, to deliver 25 Gy using a single oblique antero-posterior field angled to spare the rectum and bladder. Radiation was delivered at 220 kV at 13 mA for 564 s with a dose rate of 2.66 Gy/min (Fig. 1b). Continuous quality assurance of dose output and CBCT-based targeting was performed monthly and the output factors for varying field sizes were independently verified during commissioning, including for small field geometry.

### Animal treatments

To induce prostate regression (androgen-deprivation), male mice (8-weeks-old) were surgically castrated by standard technique. Mice were anesthetized with ketamine (100 mg/kg) and xylazine (10 mg/kg) and a 1 cm scrotal incision was made. Each testicle was individually pulled through the incision until the testicular fat pad was visualized. The vas deferens, testicular artery and veins were ligated with two silk ties and the testicle and epididymis were removed by transecting the structures with scissors in-between the ties. This was repeated for the contralateral testicle. The mice were observed for 4 weeks prior to any further interventions to allow complete regression of the prostate as previously described^15^.

To induce prostate epithelial cell proliferation and prostate regeneration, testosterone-containing Alzet pumps (DURECT Corporation, USA) that continuously deliver testosterone (Sigma Aldrich, USA) at a rate of 1.875 μg/h to maintain physiologic serums levels of testosterone^27^ were subcutaneously inserted into male mice 4-weeks post castration or as indicated (Fig. 2a).

### Assessment of prostate regeneration

To assess luminal epithelial cell proliferation in regenerating prostates, anterior prostate lobes were freshly dissected, digested into single cell suspensions, and analyzed by flow cytometry as previously described^28,29^. The following gating strategy was used to select for Ki67^+^ proliferating luminal epithelial cells: fixable viability dye eFluor 506 + (BD Biosciences, USA), CD45^−^, Ter119^−^, EPCAM^+^, CD24^+^, CD49f^−^, Ki67^+^ (all from Biolegend, USA). Non-castrated prostate tissue was used as a control, as previous published studies have established the rates of Ki67^+^ prostate epithelial cells with this technique at ~ 3%^29^.

To quantify prostate regeneration, anterior lobes were dissected, weighed, digested into single cell suspensions, and analyzed by flow cytometry as described above. Using the gating strategy described above, epithelial cells were the total EPCAM^+^ fraction, luminal cells were the EPCAM^+^ subset expressing CD24^+^, and basal cells were the EPCAM^+^ subset expressing CD49f^+^^16^. As the anterior prostate lobes are mirrored bilateral structures, only one lobe was targeted for irradiation, while the contralateral lobe was spared. Since in each mouse one anterior prostate lobe was irradiated, while the contralateral lobe was not, all experimental values were internally normalized to the control contralateral non-irradiated lobe.

### Statistical analysis

Data analysis was performed using Prism 8.0 (Graphpad, USA). All multi-group comparisons were performed using one-way ANOVA followed by Tukey’s multiple comparison test, with p values < 0.05 considered statistically significant. All data are represented as mean with standard error of the mean (SEM).

## Supplementary Information

Supplementary Legend.

Supplementary Figure S1.

## Data Availability

Not applicable as no datasets were analyzed in this study.
