# Supplementary material for: Using CT-guided stereotactic prostate radiation therapy (CT-SPRT) to assess sustained murine prostate ablation
Source: Sci Rep. 2021 Mar 22;11:6571. doi: 10.1038/s41598-021-86067-8 (PMC7985301; doi:10.1038/s41598-021-86067-8)
Supplement: Supplementary file 1 — Supplementary Legend. [file 41598_2021_86067_MOESM1_ESM.docx]

**Supplementary Fig. 1 –** Testosterone-pulse prior to CT-SPRT decreases prostate-regeneration. (A) Gross dissection of castrate anterior prostate lobes one month post *Condition 2* (irradiated lobe Left, contralateral non-irradiated control lobe Right); (B) gross dissection of castrate anterior lobes post *Condition 3* (irradiated lobe Left, contralateral non-irradiated control lobe Right). Scale bars, 5mm.
