# Supplementary material for: Using CT-guided stereotactic prostate radiation therapy (CT-SPRT) to assess sustained murine prostate ablation
Source: Sci Rep. 2021 Mar 22;11:6571. doi: 10.1038/s41598-021-86067-8 (PMC7985301; doi:10.1038/s41598-021-86067-8)
Supplement: Supplementary file 2 — Supplementary Figure S1. [file 41598_2021_86067_MOESM2_ESM.pdf]

Supplementary Fig. 1

a

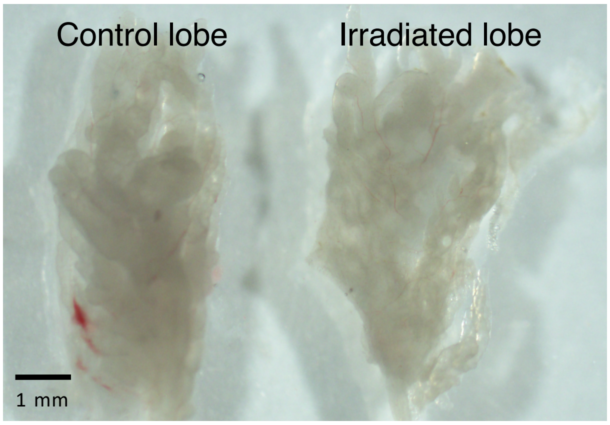

Castration/CT-SPRT/Regeneration
(Condition 2)

b

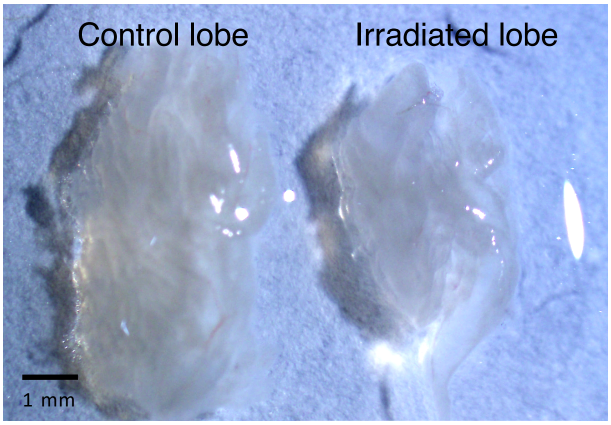

Castration/Tpulse/CT-SPRT/Regeneration
(Condition 3)
